# Screening of Serum Alkaline Phosphatase and Phosphate Helps Early Detection of Metabolic Bone Disease in Extremely Low Birth Weight Infants

**DOI:** 10.3389/fped.2021.642158

**Published:** 2021-04-22

**Authors:** Hui Zhang, Qiong Jia, Meihua Piao, Yanmei Chang, Jinghui Zhang, Xiaomei Tong, Tongyan Han

**Affiliations:** Department of Pediatrics, Peking University Third Hospital, Beijing, China

**Keywords:** metabolic bone disease, alkaline phosphatase, phosphate, extremely low birth weight infants, preterm infants

## Abstract

**Background:** Extremely low birth weight (ELBW, <1,000 g) infants have a high risk of metabolic bone disease (MBD). Because of the late appearance of radiological signs, diagnosis of MBD in ELBW infants might be delayed, and its prevalence underestimated in this group of patients. This study adopted serial screening of serum alkaline phosphatase (ALP) and phosphate (P) of ELBW infants to determine whether such screening is helpful for the early detection of MBD.

**Materials and Methods:** We performed a retrospective study of preterm infants with a gestational age ≤ 31 weeks and birth weight <1,000 g. MBD was absent (ALP ≤500 IU/L), mild (ALP >500 IU/L, P ≥4.5 mg/dL), and severe (ALP >500 IU/L, P <4.5 mg/dL); MBD was divided into early MBD (≤4 weeks after birth) and late MBD (>4 weeks after birth) according to the time of onset.

**Results:** A total of 142 ELBW infants were included, with a median gestational age of 28.1 (26.5–29.7) weeks and a median birth weight of 875 (818–950) g. Seventy-three cases of MBD were diagnosed, and the total prevalence was 51.4% (mild MBD, 10.6%; and severe MBD, 40.8%). Male sex, breastfeeding, and sepsis would increase the risk of severe MBD. Most MBD in ELBW infants occurred at 3–4 weeks after birth. Sixty-two percent (45/73) of infants were diagnosed as having early MBD, which are diagnosed earlier than late MBD [24 (21–26) vs. 39 (36–41), *t* = −7.161; *P* < 0.001]. Male sex [odds ratio (OR), 2.86; 95% confidence interval (CI), 1.07–7.64; *P* = 0.036], initial high ALP levels (OR, 1.02; 95% CI, 1.01–1.03; *P* < 0.001), and breastfeeding (OR, 5.97; 95% CI, 1.01–25.12; *P* = 0.049) are independent risk factors for the development of early MBD.

**Conclusion:** The risk of MBD among ELBW infants is very high. Most cases occurred early and were severe. Male sex, initial high ALP levels, and breastfeeding are closely related to the increased risk of early MBD. Serial screening of serum ALP and P helps early detection of MBD; it is recommended to start biochemical screening for ELBW infants 2 weeks after birth and monitor their biochemical markers weekly.

## Introduction

Metabolic bone disease (MBD) in premature infants is featured as reduction in bone mineral content and decrease in the number of bone trabeculae caused by insufficient calcium and phosphorus reserves and inadequate calcium and phosphorus intake after birth. It is characterized by hypophosphatemia, elevated serum alkaline phosphatase (ALP) levels, and imaging features of bone demineralization (1, 2). Severe MBD in preterm infants can lead to complications such as impaired respiratory function, rickets, pathological fractures, and growth retardation (3–5). Active nutrition managements for preterm infants in the past 20 years include optimizing total parenteral nutrition (TPN), shortening the duration of TPN, early strengthening enteral nutrition, and vitamin D supplementation. These measures seem to improve bone mineralization results, but MBD is still common in preterm infants, especially among infants with extremely low birth weight (ELBW, <1,000 g), with a reported prevalence of 30–52% in recent years (1, 6, 7). This is due to the fact that up to 80% of the mineral accumulation mainly occurs in the last 3 months of pregnancy. Other factors, such as the poor tolerability of enteral feeding after birth, TPN >4 weeks, and exposure to drugs such as diuretics and glucocorticoids, could also lead to increased risk of ELBW infants suffering from MBD (1, 2).

Prevention and early diagnosis are key to successful treatment of premature infants with MBD (5). As MBD is mostly asymptomatic, its diagnosis basically relies on radiological examination or biochemical screening. Radiological bone changes are a late sign, and biochemical abnormalities are already obvious usually before the appearance of radiological signs. Therefore, the serial investigation of serum biochemical markers might be more helpful for the early detection of bone mineral deficiency (8). At present, the most commonly used biochemical screening indicators for MBD in clinical practice are serum ALP and phosphate (P) (9). A prospective study showed that serum ALP >500 IU/L indicates osteoporosis, (sensitivity 100% and specificity 80.77%), which might be used as a reliable biomarker to predict the occurrence of poor bone mineralization in premature infants, especially ELBW infants (10). Another study evaluated the bone densities of premature infants with a gestational age ≤ 31 weeks and a birth weight ≤ 1,500 g at discharge, and it was suggested that ALP of 500 U/L and P of 4.5 mg/dL are the best cutoff values for the diagnosis and classification of MBD in preterm infants (11). MBD in preterm infants usually develops between 3 and 12 weeks after birth, and most cases occur between 4 and 8 weeks after birth (1, 12). Because postnatal ALP levels decrease with the increase with gestational age and birth weight (10, 13), the initial ALP level of ELBW infants may be high, leading to an earlier onset of MBD.

Unfortunately, currently, there is no unified MBD biochemical screening protocol. The American Academy of Pediatrics recommends in a clinical report that all very low birth weight (<1,500 g) infants to undergo biochemical screening 4–5 weeks after birth (14); retrospective studies by Chin et al. (15) found that 80.6% of preterm infants with a gestational age of <32 weeks had undergone MBD biochemical screening, with an average age of 36.6 days for the first screening. Therefore, premature infants with MBD might be discovered relatively late, and the prevalence may be underestimated, especially in ELBW infants, resulting in the missing of the best opportunity for early nutritional intervention and treatment.

This study retrospectively analyzed the serum ALP and P levels of ELBW infants monitored weekly or biweekly within 8 weeks after birth, to reveal the prevalence and peak incidence of MBD in ELBW infants, explore whether early biochemical screening would help in the early detection of MBD, and elucidate the risk factors associated with the early onset of MBD ( ≤ 4 weeks after birth).

## Materials and Methods

We conducted a retrospective study on the medical records of premature infants hospitalized in the neonatal intensive care unit (NICU) of Peking University Third Hospital from January 2014 to December 2019. This study was approved by Peking University Third Hospital Medical Science Research Ethics Committee (approval no. M2019162).

Inclusion criteria were as follows: (1) gestational age ≤31 weeks, (2) birth weight <1,000 g, and (3) serial screening of serum ALP and P weekly or biweekly after birth, until the infant was 8 weeks old or discharged, whichever is earlier. Those with severe congenital malformations or who abandoned treatment before 8 weeks, transferred to another hospital for treatment, died, or had incomplete data were excluded from the study.

The combination of biochemical screening of serum ALP and P can significantly improve the sensitivity and specificity in the diagnosis of MBD (5). Since January 2014, the MBD screening program for premature infants has been implemented in the NICU ward of Peking University Third Hospital. It is recommended that all ELBW infants be screened for serum ALP and P for the first time within 1 week after birth, recorded as initial serum ALP and P levels, and then repeated weekly or biweekly until discharge. During the study, because most of the radiological signs of MBD would appear after 6 weeks, most infants were not routinely screened by X-rays. Dual-energy X-ray absorptiometry (DEXA) or quantitative ultrasound scanning (QUS) is helpful for the diagnosis of MBD, but they are rarely used in China because of the factors such as ionizing radiation and scarcity of instruments. Therefore, this study adopted the critical values of ALP and P reported by Figueras-Aloy et al. (11), as they were shown to have the highest correlation with bone density, to diagnose and grade MBD. MBD is divided into three categories: non-MBD (ALP ≤500 IU/L), mild (ALP >500 IU/L, P ≥4.5 mg/dL), and severe (ALP >500 IU/L, P <4.5 mg/dL). In addition, the cases of diagnosed MBD were divided into early MBD and late MBD according to the time of onset. Early MBD is defined as MBD within 4 weeks after birth, and late MBD as 4 weeks after birth.

The nutrition management plan for the prevention and treatment of MBD implemented in this study aimed to optimize TPN and achieve total enteral feeding as early as possible. Specific implementation: starting parenteral nutrition within 24 h after birth; beginning with amino acids of 1.5 g/kg per day, which was increased by 0.5 g/kg per day with a maximum amount of 3.8 g/kg per day; beginning with fat of 0.5 g/ kg/d, which was increased by 0.5 g/kg per day with a maximum amount of 3.0 g/kg per day; if there were no contraindications, start enteral feeding (10–15 mL/kg per day) within 3 h after birth; breast milk (breast milk had 35 mg of calcium, 15 mg of phosphate, 1.2 g of protein, and 67 kcal/100 mL) was preferred and increased at a rate of 10–15 mL/kg per day. When the amount of breast milk reaches 80–100 mL/kg per day, a breast milk fortifier (BEBA FM85 in Germany: 1-g sachet in 20 mL of breast milk containing 100 mg of calcium, 60 mg of phosphate, 2.62 g of protein and 85 kcal/100 mL; or Similac HM Fortifier by Abbott of the United States: 0.9-g sachet in 25 mL of breast milk containing 146 mg of calcium, 81 mg of phosphate, 2.62 g of protein and 81 kcal/100 mL) was added, and half of the breast milk was fortified in the first 3–5 days and fully strengthened later if tolerated. If breast milk was insufficient, supplement with premature infant formula (PreNan, German Nestlé containing 122 mg of calcium, 71 mg of phosphate, a protein content of 2.3 g and 84 kcal/100 mL). When enteral feeding reaches 120 mL/kg per day, stop TPN (full enteral feeding goal was 160–180 mL/kg per day); vitamin D 700 IU had been given orally every day since 8 days after birth.

Clinical data collected include demographic characteristics (birth weight, gestational age, gender, delivery mode, singleton or multiple births); prenatal factors (chorioamnionitis, prenatal steroids, pregnancy with diabetes, preeclampsia); nutrition management (TPN duration, >50% enteral feeding); drugs used (caffeine, diuretics, steroids); mechanical ventilation time; laboratory tests [determination of serum ALP, P, and calcium (Ca) by automated biochemical analyzer (Beckman AU5800)]; other diseases such as septicemia (defined as clinical features with blood culture positive), bronchopulmonary dysplasia (defined as the requirement of supplemental oxygen or mechanical respiratory support for at least 28 days), and necrotizing enterocolitis (NEC, based on the modified Bell's criteria and defined as IIa stage or higher) (16); and length of hospital stay.

### Statistical Analysis

Normally distributed data were reported as mean ± SD, and independent-sample *t*-test was used for comparison between the two groups. Non–normally distributed data were reported as median (interquartile range), and Mann–Whitney *U* was used for comparison between the two groups, and Kruskal–Wallis test was used for comparison between the three groups. Categorical data were expressed as percentage and compared by χ^2^ test or Fisher exact test analysis.

In order to compare the levels of ALP, P, and Ca at different time points within 8 weeks after birth in the non-MBD, mild, and severe groups, repeated-measures analysis of variance was used to calculate intragroup differences (time effect) and intergroup differences and group × time interaction effects. Multivariate logistic regression analyses were used to identify risk factors associated with early MBD. The data were analyzed using IBM SPSS software version 25. It was considered statistically significant when *P* < 0.05.

## Results

### Patient Population

During the study period, a total of 8,225 newborns were admitted to the NICU of Peking University Third Hospital, 232 neonates were enrolled with a gestational age of ≤31 weeks and a birth weight of <1,000 g. Ninety infants were excluded (before discharge 29 infants died, 35 infants withdraw from the NICU by parents because of being worried about the long-term poor prognosis or financial difficulty, 24 infants were transferred to other hospitals for treatment, and 2 infants had incomplete clinical data). The remaining 142 infants met the inclusion criteria, with a median gestational age of 28.1 (26.5–29.7) weeks, and a median birth weight of 875 (818–950) g. The prevalence rate of MBD was 51.4% (73/142 cases). Fifteen cases (10.6%) had mild MBD, and 58 cases (40.8%) had severe MBD.

### Patient Characteristics

There were no significant differences in gestational age, birth weight, and prenatal characteristics among the three groups (Table 1); however, male sex (35/58, 60.3 vs. 26/69, 37.7%, χ^2^ = 6.484; *P* = 0.011), breastfeeding (9/58, 15.5 vs. 2/69, 2.9%, χ^2^ = 6.342; *P* = 0.013), and sepsis (21/58, 36.2 vs. 13/69, 18.8%, χ^2^ = 4.848; *P* = 0.028) increased the risk of severe MBD.

**Table 1:** Demographic, perinatal, and clinical characteristics.

**Groups**	**MBD category (*****n*** **=** **142)**			***p***
	**None (*n* = 69)**	**Mild (*n* = 15)**	**Severe (*n* = 58)**	
Birth weight, g*	890 (830–955)	820 (750–910)	875 (810–930)	0.094
Gestational age, weeks*	28.4 (27.4–29.8)	26.3 (25.4–29.3)	28.0 (26.3–30.1)	0.073
Male**	26 (37.7)	7 (46.7)	35 (60.3)	0.039
Small for gestational age **	38 (55.1)	6 (60.0)	27 (46.6)	0.452
Cesarean section **	45 (65.2)	8 (53.3)	35 (60.3)	0.654
Multiple births**	39 (56.5)	8 (53.3)	26 (44.8)	0.417
Chorioamnionitis **	10 (14.5)	2 (13.3)	8 (13.8)	1.000
Gestational diabetes **	18 (26.1)	4 (26.7)	9 (15.5)	0.318
Preeclampsia **	31 (44.9)	6 (40.0)	23 (39.7)	0.821
Antenatal corticosteroids **	60 (87.0)	10 (66.7)	48 (82.8)	0.164
Parenteral nutrition, d*	30 (23–36)	32 (20–40)	30 (27–40)	0.171
**Enteral feeds (>50%)**				
Breast milk**	2 (2.9)	2 (13.3)	9 (15.5)	0.027
Fortified breast milk**	38 (55.1)	9 (60.0)	33 (56.9)	0.935
Premature infant formula**	29 (42.0)	4 (26.7)	16 (27.6)	0.186
Caffeine use**	42 (6.09)	11 (73.3)	37 (63.8)	0.660
Diuretic use**	11 (15.9)	4 (26.7)	10 (17.2)	0.599
Steroid use**	5 (7.2)	3 (20)	8 (13.8)	0.210
Mechanical ventilation, d*	2 (0–9)	2 (0–18)	2 (0–11)	0.875
Septicemia**	13 (18.8)	5 (33.3)	21 (36.2)	0.041
BPD**	23 (33.3)	6 (40.0)	20 (34.5)	0.886
NEC**	4 (5.8)	1 (6.7)	2 (3.4)	0.632
Hospital days, d*	58 (49–72)	63 (56–77)	64 (57–76)	0.268

Data are expressed as the median (IQR) or number (%). Comparisons were performed with *Kruskal–Wallis test or **Pearson χ^2^.

*BPD, bronchopulmonary dysplasia; IQR, interquartile range; MBD, metabolic bone disease; NEC, necrotizing enterocolitis*.

### Screening of Serum Biochemical Markers

**Figure 1:**
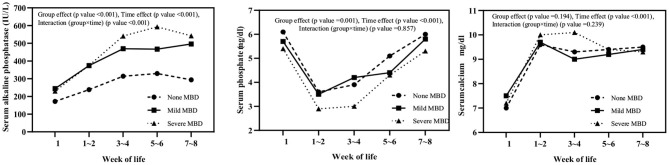
Serum alkaline phosphatase, phosphate, and calcium levels in three groups (none, mild, and severe MBD) of infants at different time points within 8 weeks after birth.

Serum ALP, phosphate, and calcium levels in three groups (non-MBD, mild, and severe) of infants at different time points within 8 weeks after birth (<1, 1–2, 3–4, 5–6, and 7–8 weeks) were monitored (Figure 1). Serum ALP in infants with mild and severe MBD was higher than that in infants without MBD, and the intergroup effect (*F* = 97.690, *P* < 0.001), time effect (*F* = 88.670, *P* < 0.001), and group × time interaction effect (*F* = 9.623, *P* < 0.001) were statistically significant; ALP levels in children with severe MBD peak at 5–6 weeks after birth (593 ± 20 IU/L). Serum P in children with severe MBD was lower than that in infants with mild and no MBD. The between-group effect (*F* = 7.001, *P* = 0.001) and time effects (*F* = 39.238, *P* < 0.001) were significantly different. The group × time interaction effect (*F* = 0.458, *P* = 0.857) is not statistically significant; the serum P levels in the three groups of children were lowest in 1–2 weeks after birth. There was a significant difference in serum Ca time effect (*F* = 52.971, *P* < 0.001) among the three groups. No differences in the intergroup effect (*F* = 1.669, *P* = 0.194) or the group × time interaction effect (*F* = 1.334, *P* = 0.239) were observed. Similarly, the serum Ca levels are lowest within 1 week after birth in all the three groups.

### Incidence of MBD in Premature Infants

Next, we investigated the incidence of MBD in preterm infants at different time points. There were no children diagnosed with MBD within 1 week of birth. The incidence of MBD at 1–2, 3–4, 5–6, and 7–8 weeks after birth were 5.6% (8/142) and 26.1% (37/142), 16.2% (23/142), and 3.5% (5/142), respectively. There was a significant difference between groups (χ^2^ = 41.168, *P* < 0.001), and the diagnosis of MBD in preterm infants peaked at 3–4 weeks after birth.

### Comparison of Clinical Features Between Early and Late MBD

Forty-five cases of early MBD (31.7%, 45/142) and 28 cases of late MBD (19.7%, 28/142) were compared (Table 2). There are more infants diagnosed with severe MBD in early MBD than in late MBD [40/45 (88.9%) vs. 18/28 (64.3%), *P* = 0.011]. ALP levels in early MBD (within 1 week after birth) are higher than those in late MBD (263 ± 75 vs. 187 ± 65 IU/L, *t* = 4.446; *P* < 0.001). Diagnosis time for early MBD is earlier than late MBD [24 (21–26) vs. 39 (36–41) days, *t* = −7.161; *P* < 0.001].

**Table 2:** Comparison of clinical characteristics between early and late MBD.

	**Early MBD (*n* = 45)**	**Late MBD (*n* = 28)**	***P***
Gestational age, weeks*	27.3 (26.3–29.8)	28.2 (26.2–30.2)	0.609
Birth weight, g*	870 (820–950)	840 (772–900)	0.103
Male**	28 (62.2)	14 (50.0)	0.304
Severe MBD**	40 (88.9)	18 (64.3)	0.011
Initial serum ALP, IU/L***	263 ± 75	187 ± 65	0.000
Initial serum P, mg/dL***	5.6 ± 0.7	5.7 ± 0.6	0.646
Time of diagnosis, d*	24 (21–26)	39 (36–41)	0.000

Data are reported as the median (IQR) or number (%) or mean ± SD. Comparisons were performed with *Kruskal–Wallis test or **Pearson χ^2^ or ***Student t-test.

*ALP, alkaline phosphatase; CI, confidence interval; IQR, interquartile range; MBD, metabolic bone disease; OR, odds ratio; P, phosphorus*.

### Analysis of Early MBD Risk Factors

Univariate logistic regression analysis showed that male sex and relatively initial high serum ALP levels increased the risk of early MBD. In addition, breastfeeding increased the risk of early MBD compared to formula milk feeding in preterm infants (Table 3). Multivariate logistic regression analysis of factors that are statistically significant showed that male sex (OR), 2.86; 95% confidence interval (CI), 1.07–7.64; *P* = 0.036], and initial high serum ALP levels (OR, 1.02; 95% CI, 1.01–1.03; *P* < 0.001) increased the risk of early MBD. Consistent with the results above, breastfeeding compared with formula feeding in preterm infants also increased the risk of early MBD (OR, 5.97; 95% CI, 1.01–25.12; *P* = 0.049) (Table 4).

**Table 3:** Univariate logistic regression analysis of early MBD risk factors.

**Factors**	**Early MBD (*n* = 45)**	**Non-MBD (*n* = 69)**	**OR**	**95% CI**	***P***
**Birth weight, g**					
≤800	10 (22.2)	12 (17.4)	1.39	0.50–3.86	0.529
801–900	17 (37.8)	27 (39.1)	1.05	0.45–2.44	0.911
901–999	18 (40.0)	30 (43.5)	Reference		
**Gestational age, weeks**					
≤28	27 (60.0)	25 (36.2)	1.67	0.66–4.24	0.282
28^+1^-30	7 (15.6)	27 (39.1)	0.40	0.13–1.23	0.111
30^+1^-31	11 (24.4)	17 (24.7)	Reference		
Male	28 (62.2)	26 (37.7)	2.72	1.25–5.91	0.011
Small for gestational age	18 (40.0)	38 (55.1)	0.54	0.25–1.16	0.117
Multiple births	19 (42.2)	39 (56.5)	0.56	0.26–1.20	0.137
**Enteral feeds (>50%) in the first 4 weeks of life**					
Breast milk	9 (20.0)	2 (2.9)	9.67	2.15–43.56	0.003
Fortified breast milk	27 (60.0)	38 (55.1)	2.35	0.96–5.77	0.062
premature infant formula	9 (20.0)	29 (42.0)	Reference		
**Treatments in the first 4 weeks of life**					
Mechanical ventilation	25 (55.6)	36 (52.2)	1.15	0.54–2.44	0.724
Steroid use	6 (13.3)	4 (5.8)	1.97	0.56–6.89	0.289
Diuretic use	6 (13.3)	10 (14.5)	0.97	0.35–2.73	0.956
Caffeine use	27 (60.0)	10 (14.5)	0.96	0.45–2.08	0.926
Initial serum ALP, IU/L	263 ± 75	177 ± 56	1.02	1.01–1.03	0.000
Initial serum P, mg/dL	5.6 ± 0.7	5.9 ± 0.5	0.88	0.68–1.11	0.314
Initial serum Ca, mg/dL	7.4 ± 0.8	7.0 ± 0.6	1.27	0.91–1.77	0.153

*Data are reported as the number (%) or mean ± SD*.

*ALP, alkaline phosphatase; Ca, calcium; CI, confidence interval; MBD, metabolic bone disease; OR, odds ratio; P, phosphorus*.

**Table 4:** Multivariate logistic regression analysis of early MBD risk factors.

**Factors**	***B***	**SE**	**Wald χ^**2**^**	**OR (95% CI)**	***P***
Male	1.05	0.50	4.41	2.86 (1.07–7.64)	0.036
Initial serum ALP, IU/L	0.02	0.01	20.92	1.02 (1.01–1.03)	0.000
**Enteral feeds (>50%) in the first 4 weeks of life**					
Breast milk	1.79	0.91	3.89	5.97 (1.01–25.12)	0.049
Fortified breast milk	1.06	0.56	3.57	2.88 (0.96–8.67)	0.059
Premature infant formula	Reference				

*ALP, alkaline phosphatase; CI, confidence interval; MBD, metabolic bone disease; OR, odds ratio*.

## Discussion

MBD is a common comorbidity of ELBW infants, yet MBD screening, diagnosis, prevention, and treatment have not been unified to date. The exact prevalence of MBD is still imprecise and may be underestimated and diagnosed late. In our study, the prevalence of MBD in ELBW infants diagnosed by serial screening of serum ALP and P levels was higher than that in most reported studies, and the onset of the disease was also relatively earlier. It was also revealed that male sex, breastfeeding, and initial high ALP levels were closely related to a higher risk of early MBD.

In our study population, the total prevalence of MBD in premature infants with gestational age ≤ 31 weeks and birth weight <1,000 g was 51.4% (73/142), with the highest incidence at 3–4 weeks after birth, and the peak incidence was higher than that in earlier reports. Retrospective studies from Viswanathan et al. (6) have shown that the prevalence of ELBW infants with a gestational age of ≤ 30 weeks by radiological examination is 30.9% (71/230), and the onset time is 58.2 ± 28 days. Lee et al. (17) reported that the incidence of radiation rickets in ELBW infants was 44%, and the diagnosis time was 48.2 ± 16.1 days. ALP is negatively correlated with gestational age and birth weight (10, 13), suggesting that the initial ALP level of ELBW infants is relatively high. In this study, the initial ALP levels of mild and severe MBD were higher than that of children without MBD. The ALP level increased gradually after birth, and the level of blood phosphorus reached the lowest point in 1–2 weeks after birth, which may cause MBD to appear early in 3–4 weeks after birth.

Shiff et al. (18) demonstrated that ELBW infants had significantly increased osteoblast activity in the first 3 weeks after birth, and the biochemical indicators involved in bone transformation were also significantly increased and continued until 10 weeks after birth. Rustico et al. (12) studies have also proved that hypophosphatemia is the earliest sign of mineral metabolism disorders, as early as 7–14 days after birth. Therefore, the time from birth to 3–4 weeks after birth may be a vital period of bone formation in premature infants, and ELBW infants during this period are more susceptible to have the complications of sepsis, liver disease, kidney disease, NEC, etc. The requirement of drug treatment (such as caffeine, diuretics, steroids, etc.) and poor tolerability of enteral feeding disturb bone mineralization and could lead to accelerated development of MBD in premature infants. Because bone mineralization can only be recognized by X-rays when reduced by 20–40%, radiological examination is unreliable in the early stages of MBD and will delay the diagnosis of MBD (5). Abdallah et al. (10) have also confirmed the effect of serial ALP levels as a biomarker for bone loss in preterm infants. In conclusion, ELBW infants should be biochemically screened from 2 weeks after birth and monitored once a week to help detect early MBD.

Severe MBD accounts for 80% (58/73) of children diagnosed with MBD. Male sex, breastfeeding, and sepsis increase the risk of severe MBD. Compared with female infants, male infants have a low renal tubular reabsorption rate and increased renal phosphorus excretion, which may be related to delayed kidney maturity, leading to the occurrence of hypophosphatemia, which is a risk factor for MBD in preterm infants (19, 20). Compared with preterm infant formula or intensive breastfeeding, breastfed preterm infants have lower phosphate levels (21); Adamkin et al. (22) have shown that the prevalence of rickets in breastfed preterm infants is 40%, and formula milk powder has reduced the prevalence to 16%; this may be due to that intensive breastfeeding or formula milk feeding provides adequate calcium and phosphorus for premature infants and helps prevent MBD. Sepsis has been shown to impair bone remodeling by stimulating osteoclast activity, reducing calcium absorption by the intestine, and increasing calcium excretion by the kidneys (2).

The monitoring of serum biochemical markers helps early detection (third week after birth) of mineral deficiencies (2). Among the children diagnosed with MBD in this study, 62% (45/73) were found within 4 weeks after birth. The median time for diagnosis of early MBD was 24 days, and that for diagnosis of late MBD diagnosis was 39 days. Early MBD is always severe, and the proportion of infants diagnosed with severe MBD is higher in early MBD than in late MBD. Early MBD is associated with male sex, increased initial ALP levels, and breastfeeding. Human milk alone may have inadequate protein and mineral content to promote optimal growth in the preterm infant because phosphate levels are lower in premature babies and breastfed infants (19–21). A lower phosphate level accompanied by initial high ALP levels and the effect of accumulation over time would affect bone mineralization, leading to the early occurrence of MBD. Because the advantages of breastfeeding are well-established in the protection against sepsis and NEC (2, 7), we need to improve fortification and mineral intakes by human milk fortifier. Unfortunately, the optimal time point at which the fortifier should be added to human milk for promoting growth in preterm infants has not been decided yet. The common practice is to start fortification when the amount of breast milk reaches 100 mL/kg per day (19, 23, 24). However, in clinical scenario, fortification is delayed because of clinicians' concern about feed intolerance and the risk of NEC. Thanigainathan et al. (24) showed that early fortification of human milk (started at <100 mL/kg per day enteral feed volume or <7 days' postnatal age) compared to late fortification (started at ≥100 mL/kg per day feeds or ≥7 days postnatal age) may have little or no effect to promote growth in preterm infants. Further large-scale trials would be needed to determine the optimal time of fortification.

In infants diagnosed with MBD during hospitalization, it is still controversial whether additional calcium, phosphorus, and vitamin D are required in parenteral nutrition and enteral nutrition. Chin and colleagues have found that in premature infants with MBD, ALP level improved after treatment with phosphorus supplementation, with increased serum P levels at the meantime (15). Chinoy and colleagues have shown that supplementing phosphorus in premature infants with MBD can reduce serum ionized calcium levels, cause secondary hyperparathyroidism, and aggravate MBD (14). Pelegano et al. (25) have shown that calcium and phosphorus have poor solubility in parenteral nutrition, which may lead to metabolic acidosis and hypercalciuria, limiting the increase in mineral supply through parenteral nutrition. The recommended vitamin D supplementation for the prevention of premature infants with MBD ranges from 200 IU/d (26) to 1,000 IU/d (27). During our study, the NICU ward implemented optimized TPN and achieved total enteral feeding as early as possible. Enteral feeding is recommended for intensive breastfeeding or formula milk for preterm infants, and oral vitamin D (700 IU/d) treatment is recommended to start 8 days after birth. The next step requires a prospective clinical study of the best nutritional intervention protocol for children with early MBD, so that children with MBD could receive early treatment to reduce the incidence of complications.

Serum ALP and P are the most commonly used predictors of poor bone mineral formation in premature infants, but there is no consensus for the cutoff value of the two and the imaging signs of MBD. The cutoff value of predicted serum ALP in MBD varies, ranging from 300 IU/L (28) to 900 IU/L (29), with the cutoff value of serum P ranging from 3.6 (30) to 5.6 mg/dL (29). For example, a retrospective study showed that the sensitivity of using ALP >500 IU/L to diagnose MBD in ELBW infants was 76%, and the specificity was 66.6% (6); Hung and colleagues have reported ALP in preterm infants with gestational age <34 weeks reached over 700 IU/L at 3 weeks after birth, which has a sensitivity of 73% and a specificity of 73% in predicting full-term osteoporosis (23). In contrast, Backstrom et al. (29) have shown that ALP >900 IU/L and P <5.6 mg/dL is 100% sensitive and 70% specific for the diagnosis of MBD.

The limitations of our study are that it is a retrospective, single-center study with limited sample size. As there is no routine screening X-ray, DEXA, or QUS or other biochemical markers, such as parathyroid, gland hormones, urinary calcium, and urinary phosphorus, to evaluate bone mineralization, only serum ALP and P levels were used to diagnose and grade MBD, which may cause overdiagnosis. Despite its limitations, we have found that for high-risk groups such as ELBW infants, poor bone mineralization tends to occur earlier and is often severe. Biochemical markers should be regularly monitored for bone mineralization damage and insufficient mineral intake at early stages. After early diagnosis of MBD, follow-up management could focus on exploring the appropriate calcium and phosphorus supplements based on the reduction of calcium or phosphate and maintaining optimal proportion of calcium and phosphorus intake. Whether all these measures could be beneficial to the early treatment of MBD or reduce the short- and long-term complications of MBD are to be investigated in the future.

## Conclusion

The incidence of ELBW infants suffering from MBD is quite high, with early onset. Male sex, initial high levels of ALP, and breastfeeding are independent risk factors for early MBD. For high-risk children, serial screening of serum ALP and P is of great benefit for the early detection of MBD. Therefore, it is highly recommended to initiate biochemical screening for ELBW infants 2 weeks after birth and monitor those biochemical markers weekly. Nonetheless, clinical research is in need for better treatment plan of children with early MBD in the early stage, accompanied by the QUS, to assess bone density for follow-up, to reduce the occurrence of short- and long-term complications, and improve prognosis.

## Data Availability Statement

The raw data supporting the conclusions of this article will be made available by the authors, without undue reservation.

## Ethics Statement

The studies involving human participants were reviewed and approved by Peking University Third Hospital Medical Science Research Ethics Committee (M2019162). Written informed consent from the participants' legal guardian/next of kin was not required to participate in this study in accordance with the national legislation and the institutional requirements.

## Author Contributions

HZ and TH drafted and revised the manuscript. QJ, YC, and JZ contributed to the formal analysis and data collation of important knowledge content. MP and XT critically reviewed the manuscript. All authors contributed to the article and approved the submitted version.

## Conflict of Interest

The authors declare that the research was conducted in the absence of any commercial or financial relationships that could be construed as a potential conflict of interest.
